# Quantification of a Cardiac Biomarker in Human Serum Using Extraordinary Optical Transmission (EOT)

**DOI:** 10.1371/journal.pone.0120974

**Published:** 2015-03-16

**Authors:** Tao Ding, Minghui Hong, A. Mark Richards, Ten It Wong, Xiaodong Zhou, Chester Lee Drum

**Affiliations:** 1 Cardiovascular Research Institute, Yong Loo Lin School of Medicine, National University of Singapore, Singapore, Singapore; 2 Department of Electrical and Computer Engineering, National University of Singapore, Singapore, Singapore; 3 Institute of Materials Research Engineering, A*STAR (Agency for Science, Technology and Research), Singapore, Singapore; University of Oldenburg, GERMANY

## Abstract

Nanoimprinting lithography (NIL) is a manufacturing process that can produce macroscale surface areas with nanoscale features. In this paper, this technique is used to solve three fundamental issues for the application of localized surface plasmonic resonance (LSPR) in practical clinical measurements: assay sensitivity, chip-to-chip variance, and the ability to perform assays in human serum. Using NIL, arrays of 140 nm square features were fabricated on a sensing area of 1.5 mm x 1.5 mm with low cost. The high reproducibility of NIL allowed for the use of a one-chip, one-measurement approach with 12 individually manufactured surfaces with minimal chip-to-chip variations. To better approximate a real world setting, all chips were modified with a biocompatible, multi-component monolayer and inter-chip variability was assessed by measuring a bioanalyte standard (2.5−75 ng/ml) in the presence of a complex biofluid, human serum. In this setting, nanoimprinted LSPR chips were able to provide sufficient characteristics for a ‘low-tech’ approach to laboratory-based bioanalyte measurement, including: 1) sufficient size to interface with a common laboratory light source and detector without the need for a microscope, 2) high sensitivity in serum with a cardiac troponin limit of detection of 0.55 ng/ml, and 3) very low variability in chip manufacturing to produce a figure of merit (FOM) of 10.5. These findings drive LSPR closer to technical comparability with ELISA-based assays while preserving the unique particularities of a LSPR based sensor, suitability for multiplexing and miniaturization, and point-of-care detections.

## Introduction

The use of nanohole structures as chemical sensors has been emphasized in numerous studies since the phenomenon of extraordinary optical transmission (EOT) was reported by Ebbesen et al. in 1998 [1]. Enhanced transmission occurs when incident light impinges on a metal film patterned with arrays of sub-wavelength nanohole structures and at specific wavelengths it could be attributed to the coupling of light with Bloch-wave surface polariton (BW-SPP) and/or localized surface plasmon (LSP) [2,3]. In general, molecular adsorption in the vicinity of a metal interface alters the dielectric condition, which shifts the transmission maximum(s) in the spectrum. The underlying physics of nanohole sensors has been extensively studied and its spectrum is known to be tunable through engineering of shape, size and separation distance on a nanometer scale [4–8]. Compared to commercial surface plasmon resonance (SPR) platforms, EOT-based sensors exhibit spectroscopic bands which allow for specificity in the study of molecular interactions [9–13]. We thus present the first report of nanohole EOT plasmonics, used in human serum to measure a model cardiac biomarker, troponin I.

The typical experimental setup used in early studies visually aligns the EOT-based sensor with an optical path and focuses the light beam onto the sensor surface. A large experimental apparatus, including an optical microscope, light source, and detector, is required due to the limitations of current EOT manufacture. We thus sought to create a substantially larger experimental surface, using nanoimprinting lithography (NIL), thus allowing ease of interface with a standard fibre optic cable.

**Fig 1:**
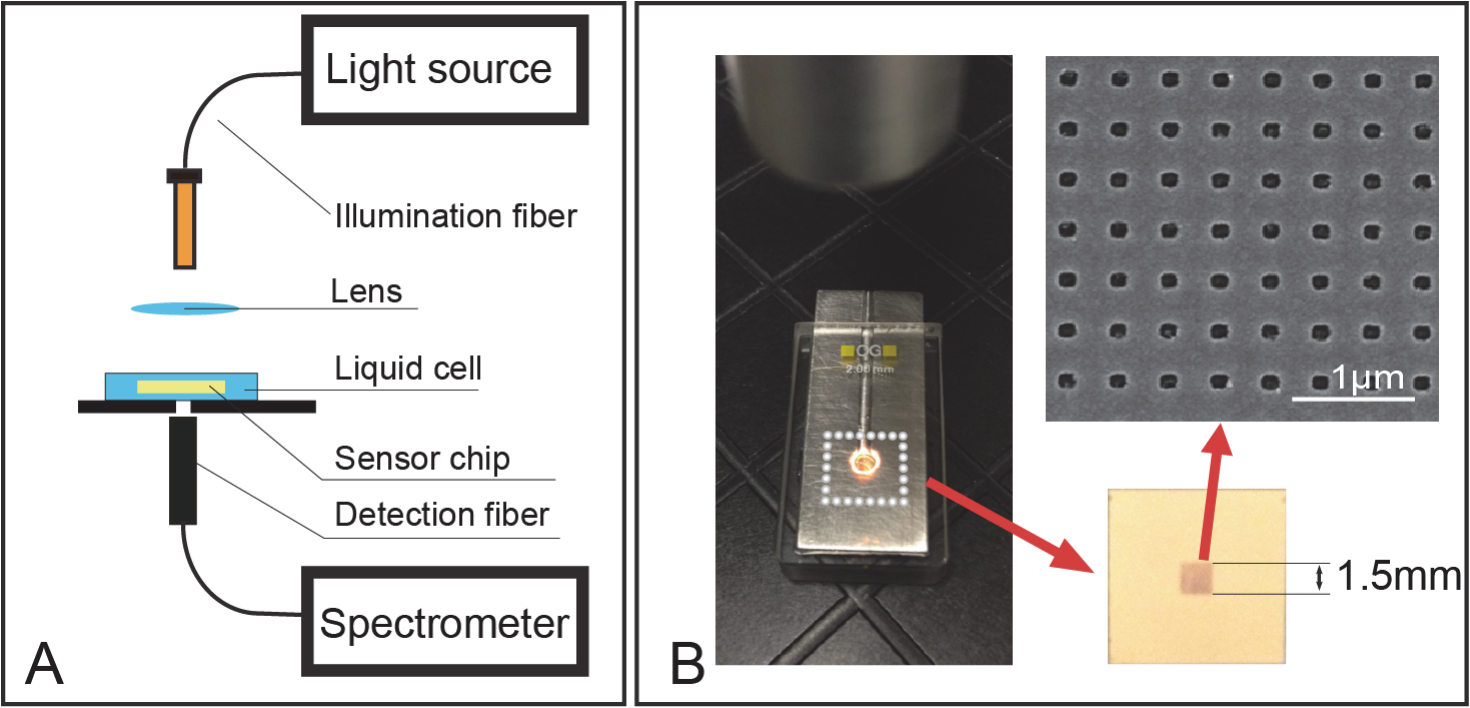
Overview of the experimental setup. A) Schematic diagram of the prototype point-of-care setup for nanohole sensor detection. B) Picture of a liquid cell during measurement. The nanohole sensor chip with a sensing area of 1.5mm X 1.5mm was inserted for detection. Inset: scanning electron micrograph of square nanoholes (square length 140 nm, pitch 400 nm) fabricated using the NIL method.

Prior work on nanohole interaction spectroscopy has focused on expensive e-beam lithography or focused ion beam lithography which are limited to dimensions of typically less than 100 microns. Large area techniques likewise suffer from poor reproducibility, in particular nanosphere lithography, interference lithography, or co-polymer templates [14,15]. For the above reasons, we adapted the newly developed method of nanoimprinting lithography (NIL) as a biosensing technology due to its millimeter fabrication scale [16], and demonstrated high reproducibility [17]. Using this novel approach, we found that sensing areas in the millimeter scale greatly enhanced a flexible linear optical detection scheme and allowed biomarker detection in a complex fluid, namely human serum, using conventional optical fibres (Fig. 1). The nanohole array’s transmission peaks are in the visible to near infrared (NIR) region, thus measurements of biomolecules in their native state can be made in an aqueous environment. The nanohole array biosensor was first characterized using computer simulation and by referencing liquids of known refractive index, followed by measuring human cardiac troponin I (cTnI) in human serum samples which is the gold standard for the diagnosis of acute myocardial infarction. For the first time, we demonstrated the robustness of large size EOT based nanohole array biosensors in a portable optical setup. Using our fabrication process, NIL nanohole chips of millimeter scale can measure cTnI in serum at a clinically relevant limit of detection (LOD) of 0.55 ng/ml with a detection time much shorter than existing technologies such as conventional ELISA. Further differentiating our NIL based detection, the nanohole surface can be easily regenerated for repeated assay use. These findings, taken together, for the first time illustrate the feasibility of nanohole array biosensors as viable substrates for industrial engineering and measurement validation within complex biofluids.

## Materials and Methods

### Sensor fabrication and data acquisition

The details of fabricating a large area nanohole sensor have been described previously [17]. Briefly, a 4” nickel mold with desired nanohole arrays repeatedly patterned in different areas was fabricated, which was realized by (1) e-beam writing of nanopillars (reversed pattern of the square nanoholes in the size of 140 nm) on the e-beam resist coated on a 4” silicon wafer, (2) electroplating of nickel on the silicon wafer with resist nanopillars, and (3) separating the nickel mold from the silicon wafer. The nickel mold with nanoholes endured repeated nanoimprinting. To mass fabricate the sensor chips, a 4” glass wafer was coated with 300 nm thick of photoresist and nanoimprinted by the nickel mold, then 5 nm of chromium for adhension and 95 nm of gold film for plasmonics was deposited on the nanoimprinted patterns, and the gold nanoholes were obtained after photoresist lift-off. The glass wafer with gold nanohole arrays was subsequently diced into individual 1 cm × 1 cm sensor chips, each with central 1.5 mm × 1.5 mm area filled with gold nanoholes for use in this study.

In the optical measurement apparatus, white light transmitted through an optical fiber bundle was collimated and illuminated on the nanohole array at a normal incidence angle. The transmitted signal was collected by a receiving optical fiber and recorded by a UV-visible spectrometer HR2000+ (Ocean optic, Dunedin, FL, USA) within a spectral range of 300–1000 nm. Each frame of transmission was collected using an acquisition time of 20 ms, and the final spectrum was produced by averaging 100 frames. Similar approaches were described previously by Im *et al*. for reducing the background noise of LSPR-based sensors [18]. All spectra were analyzed and evaluated using OriginPro 9 software (OriginLab Corporation, Northampton, MA, USA), and the position of all transmission peaks were identified using a Lorentz based method.

### Finite element simulation

We simulated the plasmonic field in our sensor using COMSOL software, which is based on the finite element method to provide total plasmonic field information, including the reflection, transmission and absorption light intensity as well as the plasmonic near-field distribution around the gold nanostructures at each wavelength. For the gold nanostructure array, only a quarter of single nanohole embedded in a square whose length is equal to half of the array pitch needs to be calculated [17]. The incident electric field E_0x_ has a magnitude of 0.5cε_0_E_0x_^2^ = P_in_∕(p∕2)^2^, which is about 4340 V∕m for incident power P_in_ of 1 nW over an area of 0.2 × 0.2 μm^2^ (for a 400 nm pitch nanohole array). As the incident wave struck the structure, the reflected power and transmitted power were calculated through the surface integration of the power flow over the surfaces, respectively. The absorbed power was computed through the volume integration of the resistive heating in the gold film (S1 Fig.).

### Sensor surface modification

Nanohole array sensor chip surfaces were cleaned in the order of isopropanol, acetone and deionized water, and dried at room temperature with nitrogen gas prior to chemical modifications. An amine-reactive self-assembly monolayer (SAM) was formed by incubating the sensor chips in ethanolic solution of 0.4 mM of 10-carboxy-1-decanethiol (Dojindo Laboratories, Japan) and 1.6 mM of 1-octanethiol (Sigma-Aldrich, MO, USA) for 12 h at room temperature, then washed thoroughly with pure ethanol and dried in room temperature. Subsequently, the sensor was incubated in a mixture of 75 mM of sulfo-N-hydroxysuccinimide (sulfo-NHS) and 15 mM of 1-ethyl-3-(3-dimethylaminopropyl) carbodiimide (EDC) (Bio-Rad, Hercules, CA, USA), to activate the carboxylic group of the SAM for 15 min. Next, 50 μl of 200 μg/ml anti-troponin antibody 560 (Hytest, Finland) solution (acetate buffer, pH 4.5) was spotted on the sensor surface and incubated for 30 min. Finally, the sensor chip was immersed in 1 M of ethanolamine-HCl solution (Bio-Rad, Hercules, CA, USA) for 15 min to deactivate the unreacted esters, followed by a rinse with deionized water and dried with nitrogen gas at room temperature.

### Sensor bulk sensitivity test and cTnI assay

Liquid with known refractive index (Cargill Inc., Cedar Grove, USA) ranging from 1.31–1.39 was deposited into the liquid cell. Subsequently, a sensor chip was inserted into the liquid cell, immersed in the refractive index standard liquid, and then aligned with the optical path where a transmission spectrum was obtained. After each measurement, the sensor chip and measurement cell were thoroughly rinsed with 5% decon solution and dried with nitrogen gas.

Human cTnI standard in serum was purchased from Phoenix Pharmaceuticals (Belmont, CA, USA) in four concentrations (75 ng/ml, 30 ng/ml, 7.5 ng/ml and 2.5 ng/ml). After the immobilization of the antibody onto the sensor chip, 100 μl of 1% BSA solution was deposited onto the surface and incubated for 15 min to block any non-specific binding. 50 μl of cTnI standard was applied to the chip surface and incubated in moisture environment for 30 min. The sensor chips were rinsed with PBS solution three times after incubation, and inserted into the measurement cell containing PBS buffer to record its transmission spectra. For regeneration of the chip, sensor chips were submerged with 50 mM Glycine-HCl (pH2) for 1 min, rinsed with PBS solution three times and measured in PBS solution.

### Surface plasmon resonance measurement

The Bio-Rad ProteOn XPR36 instrument (Haifa, Israel) was used in this study. The GLC type sensor chip consists of a glass prism coated with gold and an alginate layer with a low capacity for antibody conjugation. A detailed introduction of experimental protocol for antibody-antigen interaction can be found elsewhere [19]. In this regeneration test, briefly, the system was equilibrated with PBS-T buffer (20 mM Na-phosphate, 150 mM NaCl, and 0.05% Tween 20, pH 7.4). Antibody and cTnI standard were used as described in the cTnI assay. Three channels were activated for 5 min with a mixture of EDC (0.2 M) and sulfo-NHS (0.05 M), followed by 5 min injection of 50 μg/ml antibody 560 and 5 min injection of ethanolamine-HCl solution. Then the sensor chip was rotated by 90°and various cTnI standards at different concentrations were injected and conjugated to the antibody at interaction spots, where the response units were monitored in real time. The regeneration was performed by injecting 50 mM Glycine-HCl (pH 2) for 1 minute.

## Results and Discussion

### Transmission spectrum and Sensor characterization

**Fig 2:**
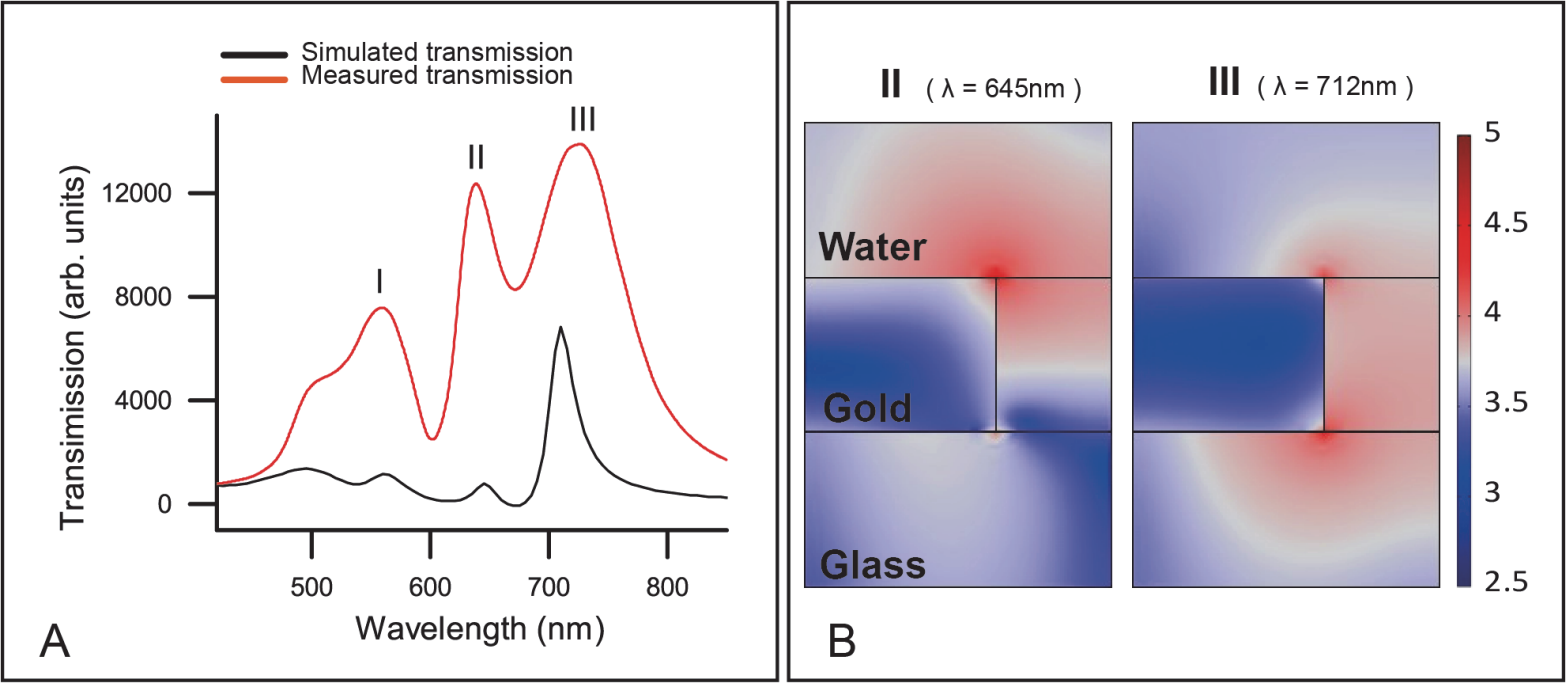
Characterization of the sensor. A) Simulated and measured transmission spectra of the nanohole array biosensor in water. B) Cross-section view of the near field distribution of band II and band III, with red color area representing strong near field distribution. Units are the optical field distribution in log scale, |E|.

To access the underlying physics of the sensor chip, numerical simulation was carried out to gain understanding of the plasmonic field distribution in aqueous environment and compared to actual measurement, with the parameters and assumption of the simulation are described in a previous study [17,20]. The simulation software COMSOL (finite element method) allows the plasmonic field construction of the periodic nanohole array with p = 400 nm, D = 150 nm and T = 100 nm and the generation of transmission spectrum, where absorption and scattering effects are also taken into account. Fig. 2A shows the superimposed calculated spectrum with the measured transmission spectrum of the nanohole sensor. The calculated transmission spectrum reveals four bands in the wavelength range of 450–850 nm: a band centered at approximately 495 nm corresponding to the interband transition of gold, and three other bands denoted as bands I-III, centered at 560 nm, 645 nm and 712 nm, respectively. Calculated bands I-III achieved very good alignment to empirically measured bands centered at 558 nm, 638 nm and 724 nm. These bands are not expected to be polarization sensitive, as the fabricated nanoholes approximate circular shapes and thus have no angular preference for the polarization state of incident light.

The plasmonic peak calculated at 645 nm was measured to be 638 nm, which is 7 nm below the simulation. This is because of two reasons. The first reason is that the hole simulated is 150 nm, while our fabricated nanoholes are 140 nm squares. According to a recent report [16], smaller nanoholes of noble metal will blue-shift the plasmonic peak. Particularly for gold, our separate simulations have found that at the wavelength of 645 nm around, for a gold nanohole array of 400 nm pitch and 100 nm thick, to increase the diameter from 100 nm to 150 nm will almost linearly increase the wavelength 20 nm (from 630–650 nm), so the actual size of 140 nm nanoholes in our experiments compared with the simulated 150 nm diameter ones will blue-shit 4 nm. Second, our fabricated gold nanohole array is 5 nm thinner than simulations. Our separate simulations show that varying the thickness of gold from 75 nm to 150 nm will linearly increase the wavelength from 635 nm to 665 nm, thus 5 nm less gold might cause the peak wavelength blue shift 2 nm. In total, our fabricated gold nanohole array should blue-shift 6 nm compared with the simulations. According to a recent study [21], for the gold nanohole array of 400 nm pitch and 150 nm diameter, a thinner gold film will reduce the intensity difference of the three peaks, and will red-shift the peak at 712 nm. This could be the reason that the band III in our experiments shifted to 724 nm, and the experimental intensities for bands I and II are higher than the simulation. The fabrication errors of the gold nanoholes might also add some discrepancy to the measured and simulated spectra.

Aside from reproducing the experimental transmission bands, COMSOL simulation directly visualizes the near field distribution of these bands in a unit cell (Fig. 2B). The unit on the right is the optical field distributions (V/m) in log-scale, and the highest intensity is around 4.7 (50119 V/m), which is about 11.5 times of field enhancement compared with the incident intensity of 4340 V/m in simulation. Bands I and III’s electromagnetic fields are predominantly located on the glass substrate, which make them less sensitive and unsuitable for sensing biomolecular interactions at the gold-water interface. In contrast, band II has a very strong LSPR component (short decay length) concentrated at the top rim of the nanoholes, thus contributes the most to the wavelength shift associated with the biosensing. Therefore, band II is chosen for analyte detection in this study.

**Fig 3:**
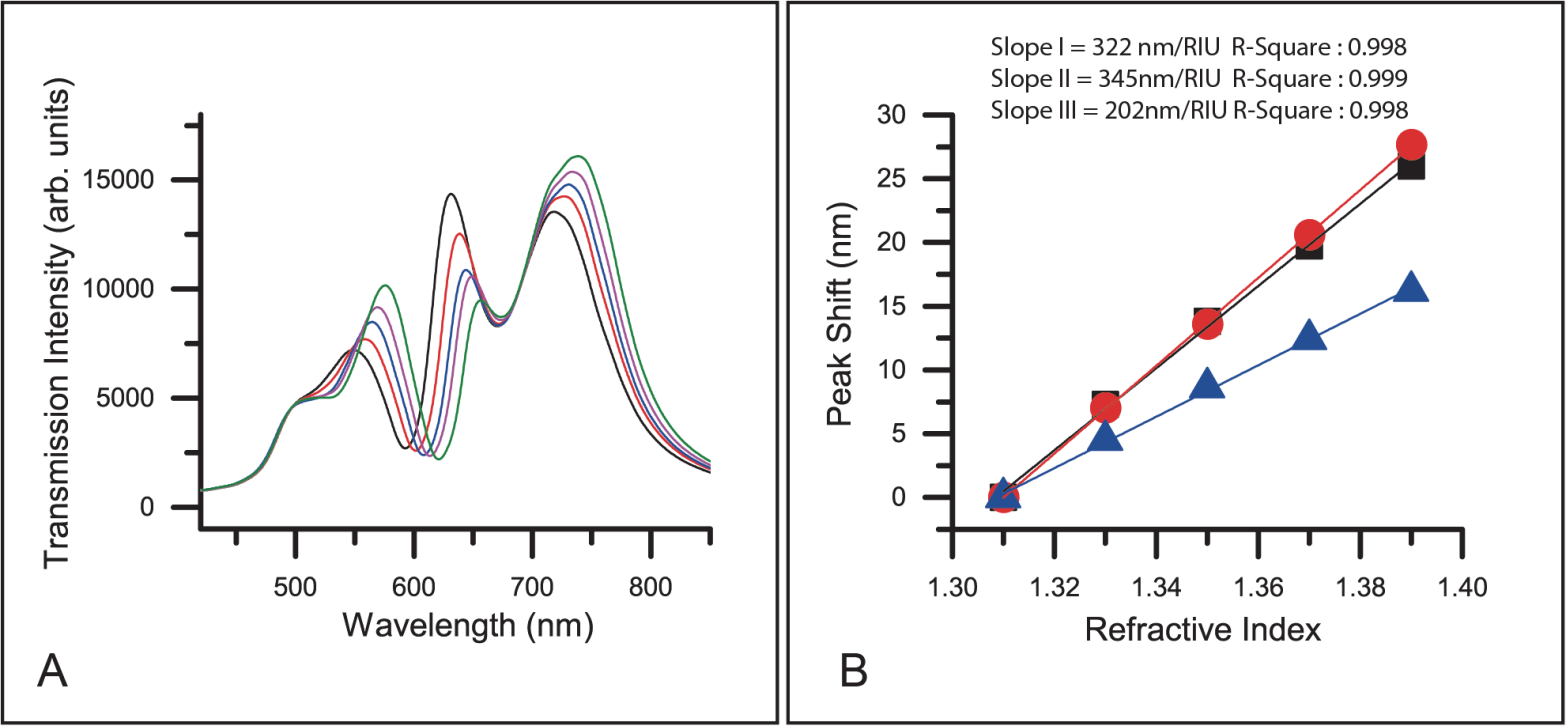
Bulk sensing performance of the sensor. A) Measured optical transmission spectra of the nanohole biosensor in refractive index standard liquid. The refractive index (RI) range is 1.31–1.39. (Black: RI 1.31, red: RI 1.33, blue: RI 1.35, purple: RI 1.37, green: RI 1.39). B) Bulk sensitivity of the three transmission bands (I-III) in visible to NIR range. (Square: band I, Circle: band II, Triangle: band III)

In order to characterize the quality of the nanohole chip and the combined performance of the optical instruments, our nanohole array sensor chips were adapted for use in a liquid cell and their transmission spectra were measured. Fig. 3A illustrates the transmission spectra of refractive index standard liquid, ranging from 1.31–1.39. Three major transmission bands were observed in the spectrum range of 400–900 nm, corresponding to bands I-III. Comparison of the spectra reveals a red-shift in band I-III, and the extent of the red-shift is in the order of band II > band I > band III. The red shift of bands I-III are summarized in Fig. 3B and the bulk sensitivities for bands I-III are determined to be 322, 345, and 202 nm/RIU, respectively. Band II has the highest sensitivity and is within our expectation as its plasmonic field is highly concentrated in the liquid, while the plasmonic fields for bands I and III are concentrated in glass (Fig. 2B).

In terms of spectral intensity, bands I and III increase with the refractive index, while band II illustrates an opposite trend. It makes sense for this kind of transmission intensity redistribution, because the increase of the reflective index on the gold surface will cause more plasmonic field to be concentrated on the gold nanohole surface (i.e., the band II) and more leaking of the plasmonic field from the glass side (where the bands I and III locate) to the liquid. As more plasmonic energy is trapped by the gold nanohole surface, the transmitted light will decrease in band II and, conversely, increase in bands I and III, as plotted in Fig. 3A. *Xiang et al*., also verified this phenomenon, noting that nanoholes plotted by extinction in transmission show an increase in peak II with liquid of higher refractive index [11].

### Performance of human cTnI assay

**Fig 4:**
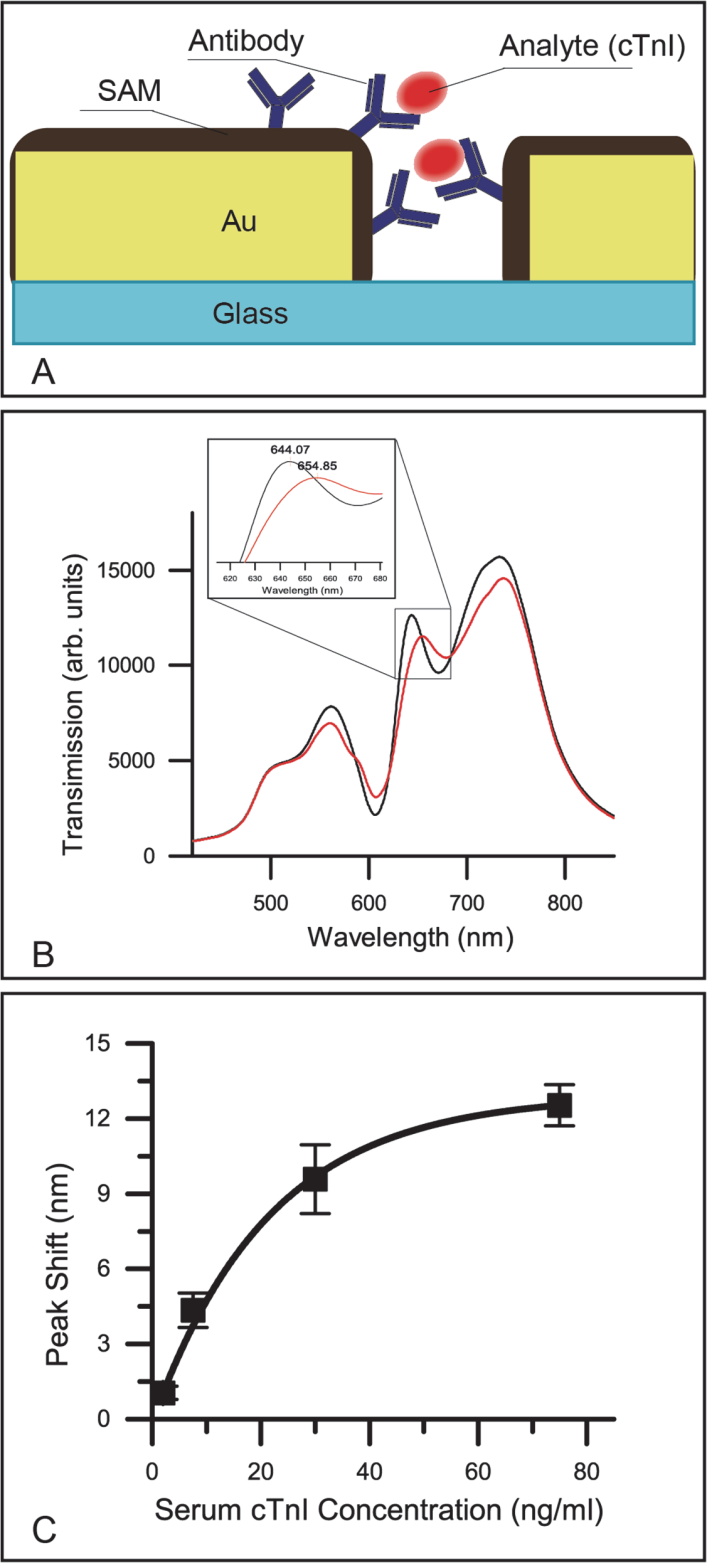
Detection of cTnI in serum. A) Schematic diagram of the analyte detection of the nanohole biosensor surface. B) Transmission spectra of the nanohole sensor before (black line) and after (red line) the measurement of 30 ng/ml human cardiac troponin in serum. Inset shows the close view of band II. C) The wavelength shift for band II at troponin concentrations of 2.5 ng/ml, 7.5 ng/ml, 30 ng/ml and 75 ng/ml (n = 3 chips for each measurement with error bars representing standard deviation)

We further characterized the robustness of the sensor and the optical detection system using human cTnI in serum. For quality assurance, we compared the band II position of our nanohole array biosensor with a bare gold surface. A red-shift of 4.1 ± 0.2 nm was observed after the amine-coupling step, which indicates that the antibody conjugation was successful (S2 Fig.). As illustrated in Fig. 4A and 4B, the observable transmission spectra directly reflect the amount of cardiac troponin molecules on the functionalized chip surface. The band shifts linearly with the troponin level at low concentrations and fits well to a binding isotherm with an R^2^ value of 0.995 and showing the beginning of saturation at 30 ng/ml (Fig. 4C). We used the measured spectra to further characterize the integrated system’s performance, with critical performance indicator analyzed to be Full Half-Magnitude Width (FHMW) = 32.84 nm, Figure of merit (FOM) = 10.5, σ of signal = 0.017 nm (FOM is calculated as resonance shift in nanometers as a function of change in refractive index, normalized by line width), Signal to noise ratio = 256 (at 7.5 ng/ml troponin in serum), and the LOD is estimated as 0.55 ng/ml. (i.e. The σ of the experimental data is the noise of the measurement. A wavelength shift of three times the standard deviation can be used in the standard curve to determine LOD of the sensor.)

We contrast our specific molecular sensing with the bulk fluid sensing seen in Fig. 2A, wherein the nanohole sensor is submerged in water and four bands were observed in the spectrum. Specifically, the interband transition band centered at approx. 495 nm and transmission bands I-III arise from the plasmonic effect of the structured Au film. When submerged in bulk liquid with increasing refractive index, bands I-III are monotonically red-shifted. This is in contrast to the spectrum observed in the molecular biosensing scenario (Fig. 4A), wherein only bands II and III had observable red-shift, and band I remained largely unchanged. This difference indicates that the shift of band I is driven by the surface plasmon polariton (SPP) mode that possess a long sensing distance. In a recent study on nanohole film of similar dimension, Schwind *et al*. associated SPP with a maximum extinction at approximately 560 nm [22], which is in agreement to our finding. Likewise, through our computational simulation, bands II and III are associated predominately with a localized SPR effect. In concordance with the simulation, the spatial distribution of the electric field in band II is highly suitable for biosensing as its sensing volume is concentrated at the gold-liquid interface, where the analyte is bound.

In our biosensing applications, we observed a relatively large band shift (Δλ) in the analyte capturing step when compared to the attachment of the capturing molecule (antibody conjugation). Compared with Fig. 3A, the intensities of all bands drop due to the surface modification (such as the cTnI antibody and cTnI layer), causing optical scattering and absorption. But the magnitude of band II is preferentially decreased as it red-shifts with biological molecule accumulation on the surface (Fig. 4A). Both observations are consistent with a recent study on another nanohole array sensor, where the Δλ reported for the analyte capturing step was 3.5 times larger, and accompanied by a significant drop in band intensity [23]. With respect to further optimization in sensor linearity, this non-linear effect between molecular mass and the transmission band shift at high analyte level should be further investigated. Nevertheless, our current technology reproducibly detects cTnI levels in serum samples, where the use of 12 individually modified chips shows the level of consistency in both chip manufacturing and chemical modification. We obtained a very good signal-to-noise ratio of 256 at analyte levels of 7.5 ng/ml, as well as a lower detection limit of 0.55 ng/ml, which is relevant for the diagnosis of acute myocardial infraction in clinical settings. It is well established that once a cardiac infarction has begun, time to intervention is one of the most important factors in keeping the heart muscle alive [24,25]. Attributes such as small device footprint, low cost, quick detection, and realistic detection range have made our nanoimprinted nanohole sensor a very attractive technological platform for biomarker quantification in medical practice. Likewise, clinically diagnostic troponin I concentrations are usually in the low single digit ng/mL range, with emerging interest in ultra-high sensitivity tests that push detection into the picogram range [26]. To address this future need, several of the inherent properties of a nanohole assay may be leveraged for increased sensitivity [27], through integrating the optical signal for a longer time, introducing spectral analysis to take overall transmission amplitude into account [28], or amplifying the wavelength shift with a sandwich assay [29,30].

### Regeneration of the sensor

**Fig 5:**
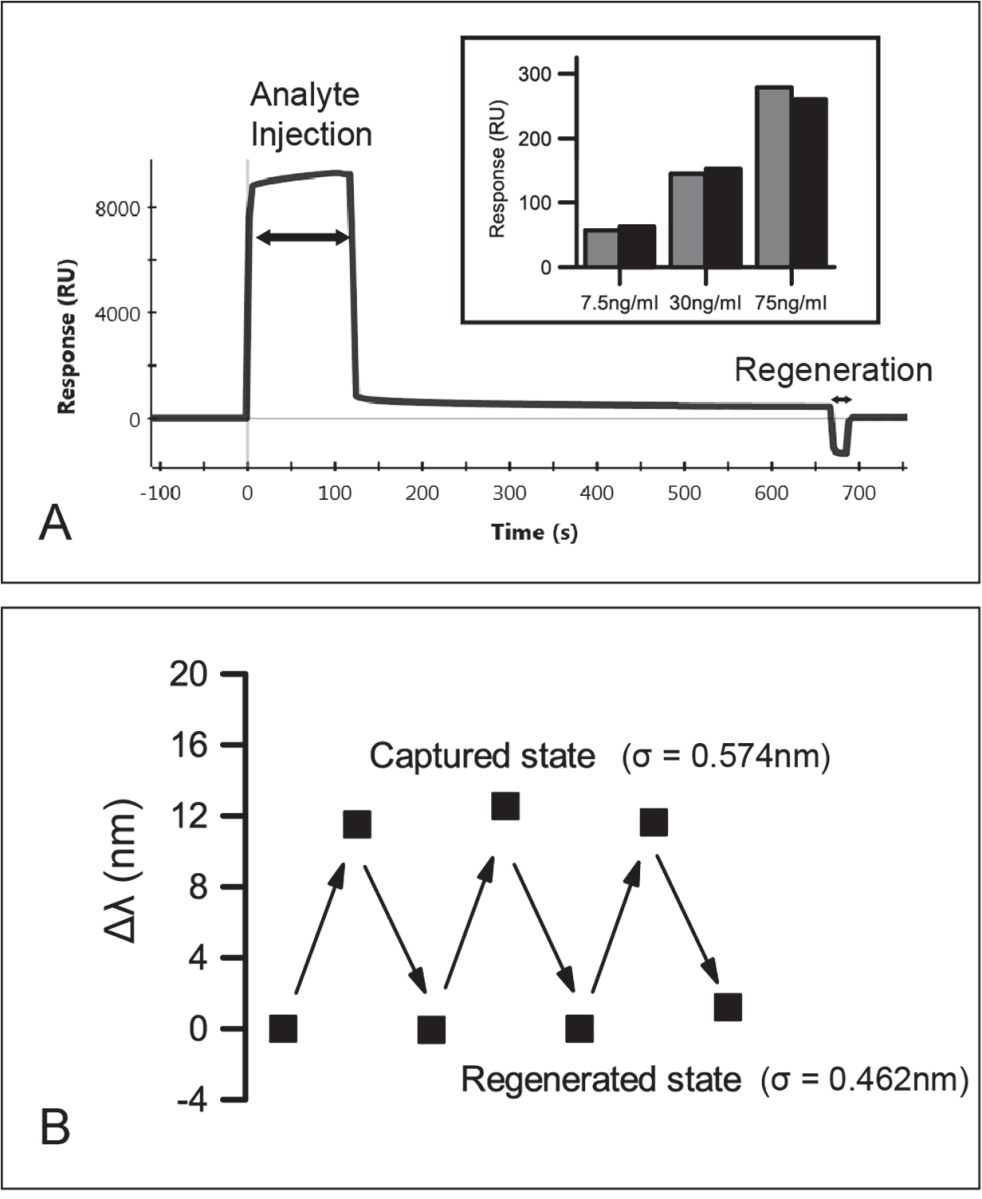
Regeneration of the sensor. A) SPR sensorgram of analyte injection (cTnI) and regeneration step. Inset: serial measurements of various cTnI in serum. Grey bar: initial measurement. Black bar: measured with regenerated surface. B) Band shift observed in regeneration test for a LSPR chip (σ = standard deviation of peak position)

The regeneration condition test was carried out using a Biorad Proteon SPR machine (Biorad). The same antibody (ab560) was conjugated onto commercial SPR chips and the increase in the response unit indicates successful conjugation (S3 Fig.). Subsequently, 75 ng/ml troponin in serum was injected onto the surface, with the sensorgram shown in Fig. 5A. An elevated response level after the cTnI injection step is observed, which indicates the capture of cTnI. Gradual dissociation of cTnI can be observed as the response decrease between 120–660 s, due to the fact that PBST (1xPBS, 0.05% tween 20) solution was flown over the chip surface. After 1 min injection of the regeneration solution, the response unit became close to 0, which was an indication of complete dissociation of cTnI from the chip surface. The inset of Fig. 5A shows the repeated engagement of cTnI sample onto the chip surface, where a highly consistent SPR response is observed and further assures the complete regeneration and the viability of the surface. We therefore applied identical regeneration conditions to the nanohole sensor (Fig. 5B), and observed similar results to the SPR study with serum cTnI concentrations at 30 ng/ml. After immersing the chip into glycine solution for 1 min, the regeneration of the chip was evident, as the position of band II shifted back approximately to its initial state. We obtained very good reproducibility of analyte detection over three consecutive regeneration and measurement rounds, showing that the regeneration process caused negligible damage to the chip surface.

SPR is the gold standard for the kinetic study of biomolecular interactions. It represents a direct and rapid method to establish regeneration conditions for bioassays based on molecular recognition. Nanofabricated LSPR chips can be modified similarly to commercial SPR chips and the mass on the surface can be monitored quantitatively in real time. Illustrated in our example here, the retaining of capturing capacity and complete disassociation of the analyte are both evident by accessing the response unit. Moreover, by combining our experimental setup with a commercially available high throughput platform, such as the BIORAD Proteon 36, multiple regeneration conditions can be tested at the same time, accelerating the optimization of solution strength and regeneration time. At present, based on the technological platform of high-sensitivity ELISA assay, diagnostic protocols for accessing patients with chest pain are well established and validated [26,31]. Due to the regenerability of these chips through multiple assay cycles, we anticipate our detection scheme may find use in point-of-care diagnostics or in situations that require non-endpoint assays, such as continuous monitoring.

### Practical considerations

We extensively study the EOT based nanohole array biosensors both theoretically and experimentally. Earlier studies aimed at fabricating highly precise nanostructures typically involve sensor areas in the micrometer scale that use an optical microscope to align the sensor to the light path [32]. While this method is effective in understanding the properties of new sensor designs, it reduces the portability and ease of use, which are two important advantages with our LSPR based sensing platform. Recently, large (>1 cm^2^) uniformly patterned metamaterials have also been created using laser interference lithography (LIL) [33], nanosphere lithography [34] and nanoimprinting [35], which allow the deployment of a simpler optical detection scheme. Following a previous report of a high-throughput and low cost method of fabricating millimeter scale nanohole sensors [17], this study further investigates its application in a simplified optical setup more amenable for general laboratory and device integration. When submerged in water (Fig. 2A), the FWHM for band II was determined to be 32.84 nm and its FOM value is 10.5, which is comparable to the values reported for sensors with a smaller footprint [23]. The sharp and defined transmission maxima and minima observed indicate a clean and highly uniformed nanostructured surface. The high fidelity of the fabrication process is further verified by the excellent correlation between simulated and experimental peak positions.

In aqueous measurement, unwanted absorption and scattering affect the overall signal-to-noise ratio and create artifacts in the spectrum. For wavelengths shorter than 600 nm, protein absorption and particle scattering play an important role in the transmission measurement, while wavelengths longer than 900 nm could obscure underlying signals, as water absorption increases with wavelength in this region. Therefore, band II is situated in the optimum window for biosensing. The small deviation (noise level) observed in the measurement of band position is also a function of the large sensor size, as larger photon flux on each detection pixel allows shorter acquisition time, and frequent temporal averaging can be achieved to lower the noise level. Nanoimprinted LSPR surfaces are further differentiated from LSPR signals generated from gold nanoparticles in solution, because the analyte binding on a surface can be easily washed and further automated into a portable point-of-care detection with controlled fluidics, while the gold nanoparticles in solution are less amenable to treatment as a stationary phase in an engineered device.

Prior to this study, the potential of using various LSPR sensors in realtime bioanalyte detection has been well demonstrated [12,32,36]. The robustness of surface regeneration is important when undertaking serial measurements in a medical device. We illustrated here that the regeneration protocol can be developed in a commercial SPR device and applied to a nanohole sensor with similar regeneration efficiency. This method is universal and provides a quantitative measure of regeneration efficiency for a ligand–analyte pair, furthermore, the surface activities after regeneration can also be accessed. Once a robust surface chemistry is established, LSPR sensors have the potential to become a very powerful and sensitive platform for real-time bioanalyte detection, where its impact on patient care is unprecedented.

## Conclusions

Although the applications of surface plasmon resonance have achieved remarkable success in academia, their penetration into clinically important analyte measurements remains limited. For this to happen, responsive surfaces are required to be created with very high reproducibility (similar to the microelectronics industry), high signal-to-noise ratio, and in a large surface area for interfacing with standard yet simple and cost effective optics. Most importantly, they need to operate with low standard deviation in the setting of a complex biofluid such as human serum. Through the use of nanoimprinted, large surface area, nanohole array biosensor chips with a simplified light path, we have made highly reproducible and clinically relevant measurements for cTnI in human serum. Thus, although the absolute sensitivity of our approach still falls short of the most advanced ELISA based tests, we anticipate that further signal enhancements by analytical software and spectral resolution might be preferable solutions to facilitate the full potential of our unique nanoimprinted LSPR sensors to monitor multiple bioanalytes in real-time patient care.

## Supporting Information

S1 Fig A quarter of the cell used for plasmonic simulation. A normal incident plane wave from the wavelength of 400 nm to 900 nm was set up at the surface S_0_ (where a boundary pair condition was applied). A linearly polarized plane wave travelling in the—z direction was assumed to be x direction polarized. For the array, the two boundaries perpendicular to x-axis were set as perfect electric conductors (PEC), and the other two boundaries perpendicular to y-axis were set as perfect magnetic conductors (PMC). The top (glass) and bottom (air/water) layers were defined as the perfect matched layer (PML) to absorb any scattered electromagnetic waves from the nanostructure. As the incident wave struck the structure, the reflected power and transmitted power were calculated through the surface integration of the power flow over the surfaces S1 and S2, respectively. The absorbed power was computed through the volume integration of the resistive heating in the gold film.
(TIF)

S2 Fig Spectra of nanohole sensor in antibody conjugation step. Black: before antibody conjugation, band position: 640.2 nm, red: after antibody conjugation, band position: 644.4 nm.
(TIF)

S3 Fig The sensorgram of antibody conjugation in SPR experiment. A significant increase in response unit indicates the successful conjugation of antibody.
(TIF)

## References

[1] Ebbesen TW, Lezec HJ, Ghaemi HF, Thio T, Wolff PA. Extraordinary optical transmission through sub-wavelength hole arrays. Nature. 1998;391(6668):667–9. 10.1038/35570 PubMed PMID: WOS:000071982500043.

[2] Stewart ME, Mack NH, Malyarchuk V, Soares J, Lee TW, Gray SK, et al Quantitative multispectral biosensing and 1D imaging using quasi-3D plasmonic crystals. Proceedings of the National Academy of Sciences of the United States of America. 2006;103(46):17143–8. 10.1073/pnas.0606216103 PubMed PMID: WOS:000242249400016. 17085594 PMC1634412

[3] Krishnan A, Thio T, Kima TJ, Lezec HJ, Ebbesen TW, Wolff PA, et al Evanescently coupled resonance in surface plasmon enhanced transmission. Optics Communications. 2001;200(1–6):1–7. 10.1016/s0030-4018(01)01558-9 PubMed PMID: WOS:000172986600001.

[4] Coe JV, Heer JM, Teeters-Kennedy S, Tian H, Rodriguez KR. Extraordinary transmission of metal films with arrays of subwavelength holes. Annual Review of Physical Chemistry2008. p. 179–202. 10.1146/annurev.physchem.59.032607.093703 17988200

[5] Liu HT, Lalanne P. Microscopic theory of the extraordinary optical transmission. Nature. 2008;452(7188):728–31. 10.1038/nature06762 PubMed PMID: WOS:000254792500040. 18401405

[6] Kim JH, Moyer PJ. Transmission characteristics of metallic equilateral triangular nanohole arrays. Applied Physics Letters. 2006;89(12). 10.1063/1.2355468 PubMed PMID: WOS:000240680300006.

[7] Yang JC, Gao HW, Suh JY, Zhou W, Lee MH, Odom TW. Enhanced Optical Transmission Mediated by Localized Plasmons in Anisotropic, Three-Dimensional Nanohole Arrays. Nano Lett. 2010;10(8):3173–8. 10.1021/nl102078j PubMed PMID: WOS:000280728900074. 20698633 PMC2921222

[8] Garcia-Vidal FJ, Martin-Moreno L, Ebbesen TW, Kuipers L. Light passing through subwavelength apertures. Reviews of Modern Physics. 2010;82(1):729–87. 10.1103/RevModPhys.82.729 PubMed PMID: WOS:000276184000017.

[9] Valsecchi C, Brolo AG. Periodic Metallic Nanostructures as Plasmonic Chemical Sensors. Langmuir. 2013;29(19):5638–49. 10.1021/la400085r PubMed PMID: WOS:000319185100002. 23488664

[10] Shon YS, Choi HY, Guerrero MS, Kwon C. Preparation of Nanostructured Film Arrays for Transmission Localized Surface Plasmon Sensing. Plasmonics. 2009;4(2):95–105. 10.1007/s11468-009-9079-1 PubMed PMID: WOS:000266390000002.

[11] Xiang GS, Zhang N, Zhou XD. Localized Surface Plasmon Resonance Biosensing with Large Area of Gold Nanoholes Fabricated by Nanosphere Lithography. Nanoscale Research Letters. 2010;5(5):818–22. 10.1007/s11671-010-9566-5 PubMed PMID: WOS:000277203500005. 20672118 PMC2893834

[12] Ji J, O'Connell JG, Carter DJD, Larson DN. High-throughput nanohole array based system to monitor multiple binding events in real time. Analytical Chemistry. 2008;80(7):2491–8. 10.1021/ac7023206 PubMed PMID: WOS:000254593500031. 18307360

[13] Stark PRH, Halleck AE, Larson DN. Short order nanohole arrays in metals for highly sensitive probing of local indices of refraction as the basis for a highly multiplexed biosensor technology. Methods. 2005;37(1):37–47. 10.1016/j.ymeth.2005.05.006 PubMed PMID: WOS:000232945500005. 16199175

[14] Gates BD, Xu QB, Stewart M, Ryan D, Willson CG, Whitesides GM. New approaches to nanofabrication: Molding, printing, and other techniques. Chem Rev. 2005;105(4):1171–96. 10.1021/cr030076o PubMed PMID: WOS:000228412800004. 15826012

[15] Biswas A, Bayer IS, Biris AS, Wang T, Dervishi E, Faupel F. Advances in top-down and bottom-up surface nanofabrication: Techniques, applications & future prospects. Advances in Colloid and Interface Science. 2012;170(1–2):2–27. 10.1016/j.cis.2011.11.001 PubMed PMID: WOS:000301688900002. 22154364

[16] Guo LJ. Nanoimprint lithography: Methods and material requirements. Advanced Materials. 2007;19(4):495–513. 10.1002/adma.200600882 PubMed PMID: WOS:000244515200001.

[17] Wong TI, Han S, Wu L, Wang Y, Deng J, Tan CYL, et al High throughput and high yield nanofabrication of precisely designed gold nanohole arrays for fluorescence enhanced detection of biomarkers. Lab on a Chip. 2013;13(12):2405–13. 10.1039/c3lc41396a PubMed PMID: WOS:000319285500024. 23645079

[18] Im H, Sutherland JN, Maynard JA, Oh SH. Nanohole-Based Surface Plasmon Resonance Instruments with Improved Spectral Resolution Quantify a Broad Range of Antibody-Ligand Binding Kinetics. Analytical Chemistry. 2012;84(4):1941–7. 10.1021/ac300070t PubMed PMID: WOS:000300470800021. 22235895 PMC3307221

[19] Nahshol O, Bronner V, Notcovich A, Rubrecht L, Laune D, Bravman T. Parallel kinetic analysis and affinity determination of hundreds of monoclonal antibodies using the ProteOn XPR36. Anal Biochem. 2008;383(1):52–60. 10.1016/j.ab.2008.08.017 PubMed PMID: WOS:000260266200007. 18782554

[20] Wu L, Bai P, Li EP. Designing surface plasmon resonance of subwavelength hole arrays by studying absorption. Journal of the Optical Society of America B-Optical Physics. 2012;29(4):521–8. PubMed PMID: WOS:000302560200001.

[21] Wu L, Bai P, Zhou X, Li EP. Reflection and Transmission Modes in Nanohole-Array-Based Plasmonic Sensors. Ieee Photonics Journal. 2012;4(1):26–33. 10.1109/jphot.2011.2177652 PubMed PMID: WOS:000298625600002.

[22] Schwind M, Kasemo B, Zoric I. Localized and Propagating Plasmons in Metal Films with Nanoholes. Nano Lett. 2013;13(4):1743–50. 10.1021/nl400328x PubMed PMID: WOS:000317549300061. 23484456

[23] Chang TY, Huang M, Yanik AA, Tsai HY, Shi P, Aksu S, et al Large-scale plasmonic microarrays for label-free high-throughput screening. Lab on a Chip. 2011;11(21):3596–602. 10.1039/c1lc20475k PubMed PMID: WOS:000295732500004. 21901194

[24] Cannon CP, Gibson CM, Lambrew CT, Shoultz DA, Levy D, French WJ, et al Relationship of symptom-onset-to-balloon time and door-to-balloon time with mortality in patients undergoing angioplasty for acute myocardial infarction. Jama-Journal of the American Medical Association. 2000;283(22):2941–+. 10.1001/jama.283.22.2941 PubMed PMID: WOS:000087480600033. 10865271

[25] McNamara RL, Wang YF, Herrin J, Curtis JP, Bradley EH, Magid DJ, et al Effect of door-to-balloon time on mortality in patients with ST-segment elevation myocardial infarction. J Am Coll Cardiol. 2006;47(11):2180–6. 10.1016/j.jacc.2005.12.072 PubMed PMID: WOS:000237981500006. 16750682

[26] Than M, Cullen L, Aldous S, Parsonage WA, Reid CM, Greenslade J, et al 2-Hour Accelerated Diagnostic Protocol to Assess Patients With Chest Pain Symptoms Using Contemporary Troponins as the Only Biomarker The ADAPT Trial. J Am Coll Cardiol. 2012;59(23):2091–8. 10.1016/j.jacc.2012.02.035 PubMed PMID: WOS:000304594800009. 22578923

[27] Dahlin AB, Tegenfeldt JO, Hook F. Improving the instrumental resolution of sensors based on localized surface plasmon resonance. Analytical Chemistry. 2006;78(13):4416–23. 10.1021/ac0601967 PubMed PMID: WOS:000238665200027. 16808449

[28] Lin EH, Tsai WS, Lee KL, Lee MCM, Wei PK. Enhancing detection sensitivity of metallic nanostructures by resonant coupling mode and spectral integration analysis. Optics Express. 2014;22(16). 10.1364/oe.22.019621 PubMed PMID: WOS:000340714100073. 25321045

[29] Guo LH, Kim DH. LSPR biomolecular assay with high sensitivity induced by aptamer-antigen-antibody sandwich complex. Biosens Bioelectron. 2012;31(1):567–70. 10.1016/j.bios.2011.10.047 PubMed PMID: WOS:000300468400090. 22099957

[30] Sharpe JC, Mitchell JS, Lin L, Sedoglavich H, Blaikie RJ. Gold nanohole array substrates as immunobiosensors. Analytical Chemistry. 2008;80(6):2244–9. 10.1021/ac702555r PubMed PMID: WOS:000254017000053. 18288819

[31] Than M, Cullen L, Reid CM, Lim SH, Aldous S, Ardagh MW, et al A 2-h diagnostic protocol to assess patients with chest pain symptoms in the Asia-Pacific region (ASPECT): a prospective observational validation study. Lancet. 2011;377(9771):1077–84. 10.1016/s0140-6736(11)60310-3 PubMed PMID: WOS:000289035000028. 21435709

[32] Lesuffleur A, Im H, Lindquist NC, Oh SH. Periodic nanohole arrays with shape-enhanced plasmon resonance as real-time biosensors. Applied Physics Letters. 2007;90(24). 10.1063/1.2747668 PubMed PMID: WOS:000247305400093.

[33] Yang J, Luo F, Kao TS, Li X, Ho GW, Teng J, et al Design and fabrication of broadband ultralow reflectivity black Si surfaces by laser micro/nanoprocessing. Light: Science & Applications. 2014;3(7):e185.

[34] Live LS, Bolduc OR, Masson JF. Propagating Surface Plasmon Resonance on Microhole Arrays. Analytical chemistry. 2010;82(9):3780–7. 10.1021/ac100177j PubMed PMID: WOS:000277213400057. 20356057

[35] Nakamoto K, Kurita R, Niwa O. Electrochemical Surface Plasmon Resonance Measurement Based on Gold Nanohole Array Fabricated by Nanoimprinting Technique. Analytical chemistry. 2012;84(7):3187–91. 10.1021/ac203160r PubMed PMID: WOS:000302829800023. 22283116

[36] Bhagawati M, You CJ, Piehler J. Quantitative Real-Time Imaging of Protein-Protein Interactions by LSPR Detection with Micropatterned Gold Nanoparticles. Analytical chemistry. 2013;85(20):9564–71. 10.1021/ac401673e PubMed PMID: WOS:000326126600022. 24016060

