# Supplementary figures and images for: Quantification of a Cardiac Biomarker in Human Serum Using Extraordinary Optical Transmission (EOT)
Source: PLoS One. 2015 Mar 16;10(3):e0120974. doi: 10.1371/journal.pone.0120974 (PMC4361334; doi:10.1371/journal.pone.0120974)

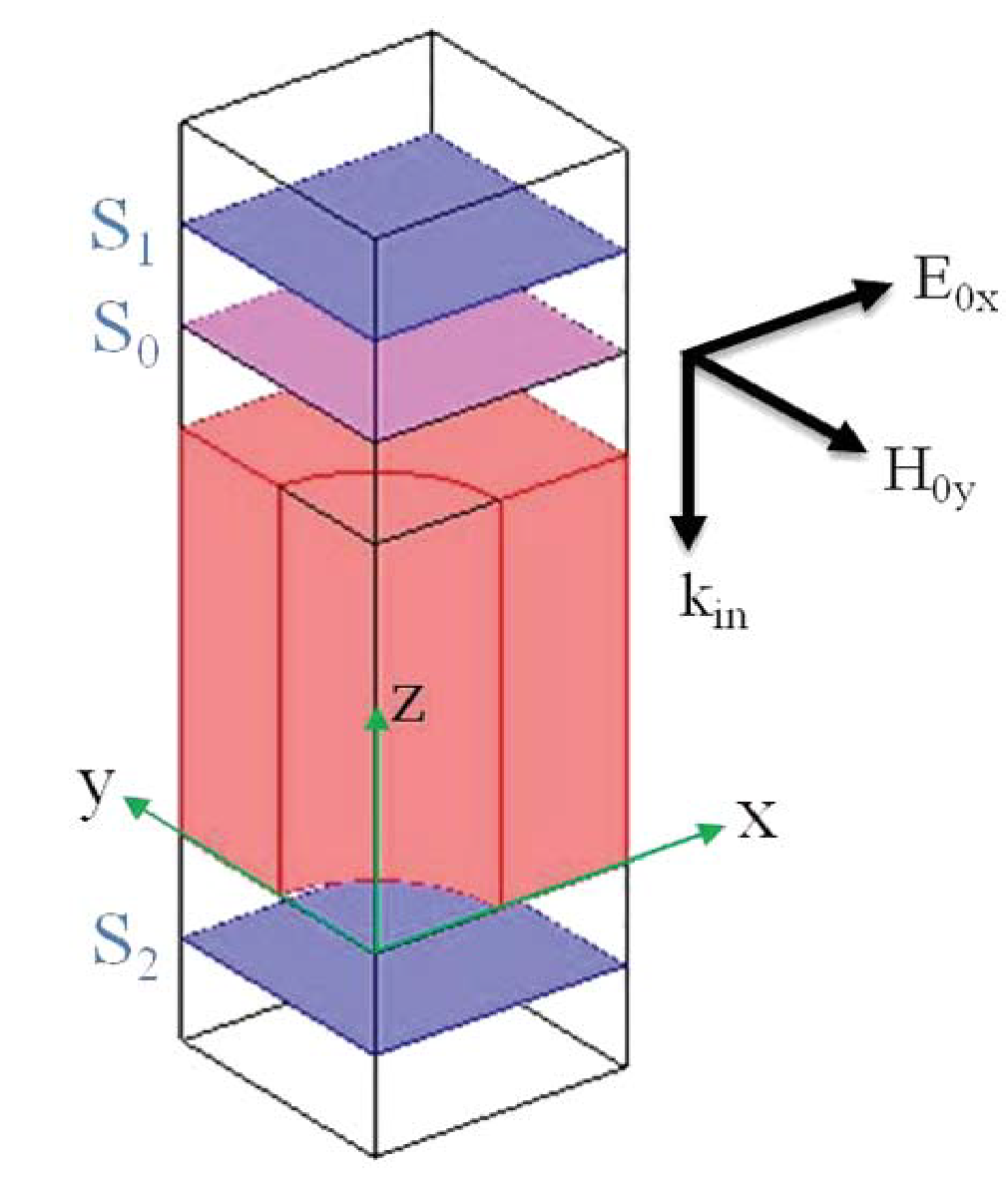

Supplement: S1 Fig — A normal incident plane wave from the wavelength of 400 nm to 900 nm was set up at the surface S0 (where a boundary pair condition was applied). A linearly polarized plane wave travelling in the—z direction was assumed to be x direction polarized. For the array, the two boundaries perpendicular to x-axis were set as perfect electric conductors (PEC), and the other two boundaries perpendicular to y-axis were set as perfect magnetic conductors (PMC). The top (glass) and bottom (air/water) layers were defined as the perfect matched layer (PML) to absorb any scattered electromagnetic waves from the nanostructure. As the incident wave struck the structure, the reflected power and transmitted power were calculated through the surface integration of the power flow over the surfaces S1 and S2, respectively. The absorbed power was computed through the volume integration of the resistive heating in the gold film. (TIF) [file pone.0120974.s001.tif]

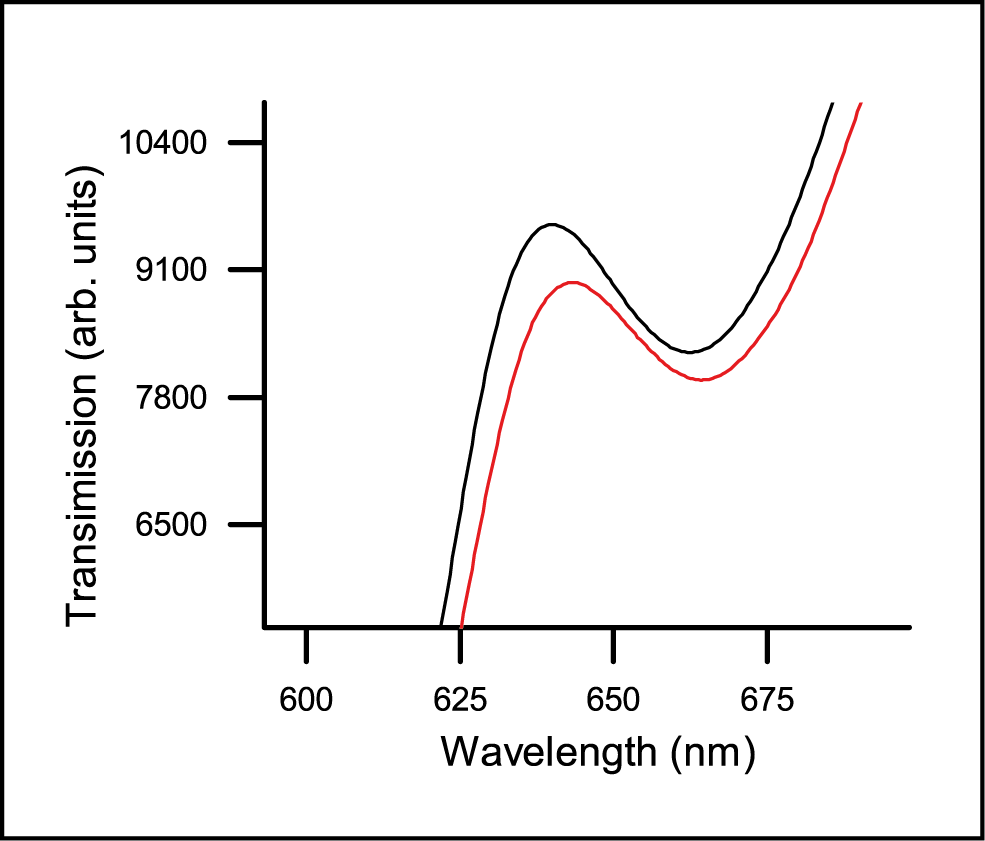

Supplement: S2 Fig — Black: before antibody conjugation, band position: 640.2 nm, red: after antibody conjugation, band position: 644.4 nm. (TIF) [file pone.0120974.s002.tif]

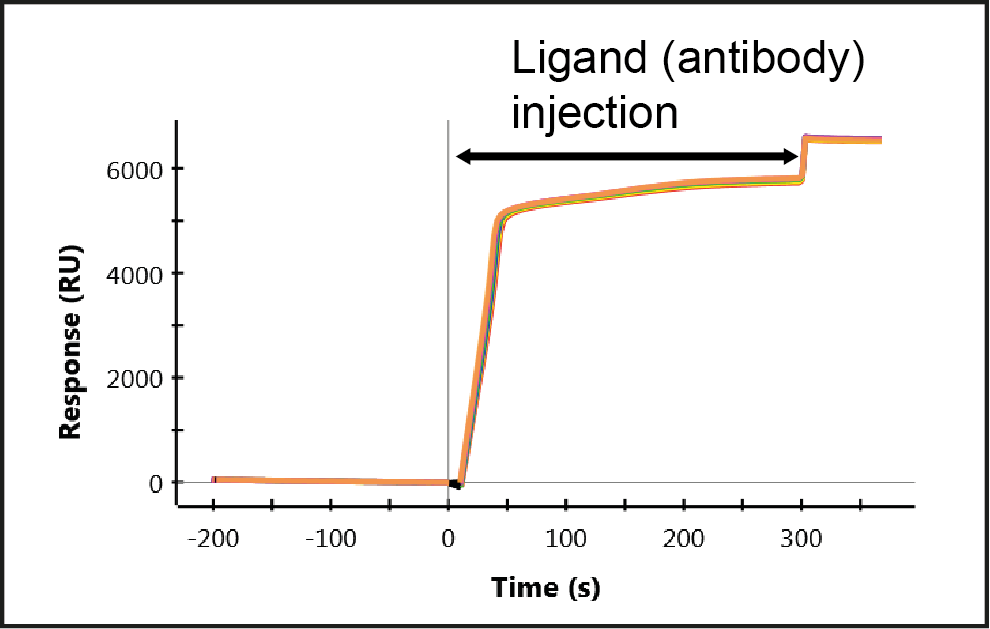

Supplement: S3 Fig — A significant increase in response unit indicates the successful conjugation of antibody. (TIF) [file pone.0120974.s003.tif]
